# Preparation and Characterization of Spray-Dried Hybrid Nanocrystal–Amorphous Solid Dispersions (HyNASDs) for Supersaturation Enhancement of a Slowly Crystallizing Drug

**DOI:** 10.3390/nano13172419

**Published:** 2023-08-25

**Authors:** Mahbubur Rahman, Keanu Radgman, James Tarabokija, Stephanie Ahmad, Ecevit Bilgili

**Affiliations:** Otto H. York Department of Chemical and Materials Engineering, New Jersey Institute of Technology, Newark, NJ 07102, USA; mahbubur1.rahman@intel.com (M.R.); kr379@njit.edu (K.R.); jst35@njit.edu (J.T.); stephanie.ahmad@infineum.com (S.A.)

**Keywords:** poorly soluble drugs, slow crystallizer, wet media milling, spray drying, drug nanoparticles, nanocomposites, amorphous solid dispersions, supersaturation

## Abstract

We prepared hybrid nanocrystal–amorphous solid dispersions (HyNASDs) to examine their supersaturation capability in the release of a poorly soluble drug, itraconazole (ITZ), a slow crystallizer during dissolution. The HyNASD formulations included a polymer (HPC: hydroxypropyl cellulose, Sol: Soluplus, or VA64: Kollidon-VA64) and a surfactant (SDS: sodium dodecyl sulfate). Additionally, the dissolution performance of the HyNASDs and ASDs was compared. To this end, wet-milled aqueous nanosuspensions containing a 1:5 ITZ:polymer mass ratio with/without SDS as well as solutions of the same ratio without SDS in dichloromethane were spray-dried. XRPD–DSC confirmed that ASDs were formed upon spray drying the solution-based feeds, whereas HyNASDs (~5–30% amorphous) were formed with the nanosuspension-based feeds. SDS aided to stabilize the ITZ nanosuspensions and increase the amorphous content in the spray-dried powders. During dissolution, up to 850% and 790% relative supersaturation values were attained by HyNASDs with and without SDS, respectively. Due to the stronger molecular interaction between ITZ–Sol than ITZ–HPC/VA64 and micellar solubilization by Sol, Sol-based HyNASDs outperformed HPC/VA64-based HyNASDs. While the ASD formulations generated greater supersaturation values (≤1670%) than HyNASDs (≤790%), this extent of supersaturation from a largely nanocrystalline formulation (HyNASD) has not been achieved before. Overall, HyNASDs could boost drug release from nanoparticle-based formulations and may render them competitive to ASDs.

## 1. Introduction

With an increasing number of poorly water-soluble drugs, aqueous solubility has become a critical issue in the pharmaceutical industry. The slow and incomplete release of poorly water-soluble drugs in the gastrointestinal tract (GI) results in slow absorption and ultimately limited bioavailability [1]. As a result, increasing the dissolution rate and solubility of poorly water-soluble drugs is of major importance. Pharmaceutical scientists have used, among various formulation approaches, drug nanocrystal-based solid dosage forms (nanocomposites) and amorphous solid dispersions (ASDs), which achieved substantial popularity [2,3,4]. While nano-size refers to drug particles below 1 µm in the prevailing pharmaceutical nanotechnology literature, pharmaceutical drug nanosuspensions with median/mean sizes in the range of 70–500 nm were prepared for the best therapeutic efficacy [5,6]. In a nanocomposite, drug nanocrystals are dispersed in the polymeric matrix as a secondary phase [2], whereas in an ASD, the drug is molecularly dispersed in a miscible, amorphous polymer to form a single phase [3]. In this paper, nanoparticles are synonymously used with nanocrystals or substantially nanocrystalline particles. Drug nanoparticles and their nanocomposites offer several advantages such as dissolution rate enhancement, improved bioavailability, elimination of food effects, etc. [7]. Hence, it is not surprising that drug nanoparticles have already been used in several marketed products [8]. On the other hand, limited improvement in the bioavailability was observed for drugs with very low aqueous solubility and high doses [9]. Such drugs, owing to the high apparent (kinetic) solubility of their amorphous form, have been formulated as ASDs. ASDs generate high supersaturation along with an enhanced dissolution rate, which results in the bioavailability enhancement of poorly soluble drugs with a high therapeutic dose [10].

Due to much larger specific surface area of the nanocrystals than the typical micron-sized drug crystals, they can significantly improve the dissolution rate of poorly water-soluble drugs [11,12] according to the classic Noyes–Whitney equation. Additionally, nanocrystals with sizes < 100 nm tend to show relatively high saturation solubility due to their high curvature, thus resulting in enhanced drug release rates. This increase in saturation solubility can be described by the Kelvin and the Ostwald–Freundlich equation [13]. The increase in apparent solubility was estimated to be ~10–15% higher than the micron-sized crystals [14], whereas a 50% increase in the apparent solubility [15] and 50–100% supersaturation generation were reported experimentally [16].

Drug nanoparticles in the form of a nanosuspension can be produced by various top-down methods such as wet stirred media milling (WSMM) [2], high-pressure homogenization [17], and microfluidization [18]. Usually, stabilizers (polymers and/or surfactants) are added to the suspensions to suppress the aggregation–growth tendency of drug nanocrystals during milling or storage [5]. They adsorb on drug nanoparticle surfaces and provide stabilization via steric, electrostatic, or electrosteric mechanisms [19]. A combination of polymers and surfactants (especially anionic surfactants) have synergistic effects on drug nanosuspension stabilization, facilitating the milling process [5,20]. A stabilizer(s) and its concentration are selected based on the intended final dosage form, physicochemical properties of the drug, stabilizer–drug interactions, and downstream processability [2,5]. If used at too low concentrations, drug nanoparticle aggregation cannot be suppressed [21]; whereas if used in excess, slower breakage of particles may occur due to viscous dampening that originates from the high concentration of the polymers [20], in addition to potential downstream processing issues [2]. Also, to achieve high drug loading in the final solid dosages, stabilizer concentration should be optimized while still achieving physical stability in the milled suspensions and good redispersibility from the drug nanocomposites and solid dosage forms [2,5].

Due to the ease of administration and patient compliance, drug nanosuspensions are usually dried and converted into nanocomposites, which are eventually incorporated into standard solid dosage forms, e.g., tablets, capsules, and sachets. Among different drying processes used for drug nanosuspensions, spray drying is one of the most cost-effective, continuous processes [2,22]. Even though significant improvement in drug release from spray-dried nanocomposites were reported in all the previous studies compared to the as-received drug micro-crystals, substantial supersaturation generation during dissolution was not reported or investigated [11,23,24,25,26] until very recently (see Table 1 for nanocomposites). Zuo et al. [27] reported up to ~50% (relative) supersaturation of fenofibrate from spray-dried nanocomposites in 0.15% sodium dodecyl sulfate (SDS) solution, which is in line with low supersaturation levels from nanocomposites [15,28]. Zuo et al. [27] also demonstrated that a significant increase in apparent solubility does not necessarily translate into high supersaturation. Hence, the true advantage of supersaturating formulations can only be discerned under supersaturating (non-sink) dissolution conditions (e.g., [27,28]).

In view of the analysis above and the studies in Table 1, it is fair to assert that drug nanocomposites appear to have limited supersaturation generation capabilities, which is the greatest impediment to their bioavailability enhancement capability and competitiveness to ASDs. On the other hand, nanocomposites have better physical stability than ASDs because the drug in ASDs can undergo severe recrystallization during storage and/or in vivo/in vitro dissolution [2,3,4,5,6]. Hence, a new approach for boosting the supersaturation capability of nanocomposites to make them competitive to ASDs is warranted. In most drug ASDs [29], a relatively high polymer concentration corresponding to low drug:polymer mass ratios from 1:1 to 1:9 was used unlike the high drug:polymer ratios in drug nanocomposites [7,30]. Recently [31], 1:1 to 1:5 drug:polymer mass ratios were used in the production of griseofulvin (GF) nanocomposites, wherein GF is a fast crystallizing poorly soluble drug [32]. This approach led to the formation of a special class of nanocomposites with notable amorphous drug content (>10%), i.e., hybrid nanocrystal–amorphous solution dispersions (HyNASDs). GF HyNASDs showed significant relative supersaturation (250%) during dissolution. The judicious choice of polymers that have relatively low aqueous viscosities even at high concentrations allowed for the preparation of drug nanosuspensions and their spray drying without any processing issues. The intrinsic recrystallization tendency of amorphous drugs in liquid media is critical to the performance of ASDs [32] and HyNASDs [31]. While 250% supersaturation from GF HyNASDs appears to be significant and is much higher than traditional nanocomposites with <100% supersaturation [31], it is much lower than the supersaturation by the ASDs (typically above 300% for GF and up to several thousand percent for other drugs). Two fundamental questions naturally emerged from [31]: can the HyNASD approach become a platform formulation strategy? Is it possible to achieve an even higher supersaturation than 250% if HyNASDs are loaded with slow crystallizers (drugs) instead of fast crystallizers like GF used in [31]?

**Table 1:** Relative supersaturation obtained from various nanocomposites, HyNASDs, and ASDs.

Drug	Material Type	Process	Formulation ^a^	% Relative Supersaturation ^b^	Ref.
Celocoxibe	Nanocomposite	Spray drying	Tween 80, dextrin	NR	[24]
Fenofibrate	Nanocomposite	Spray drying	HPMC–SDS	<50	[27]
Glyburide	Nanocomposite	Spray drying	HPMC–SDS	NR	[11]
Griseofulvin	Nanocomposite	Nanoextrusion	HPC–SDS	55	[28]
Griseofulvin	ASD	Nanoextrusion	Sol–SDS	330	[28]
Griseofulvin	Nanocomposite	Fluid bed coating	HPC–SDS	NR	[21]
Griseofulvin	Nanocomposite	Spray drying	Sol–SDS	30	[31]
Griseofulvin	HyNASD	Spray drying	Sol–SDS	250	[31]
Griseofulvin	ASD	Spray drying	Sol–SDS	360	[33]
Itraconazole	Nanocomposite	Spray drying	HPC/HPMC–SDS	NR	[23]
Itraconazole	ASD	Hot melt extrusion	HPMC	4400	[34]
Miconazole	Nanocomposite	Spray drying	HPC/HPMC–SDS	NR	[23]
Nimodipine	Nanocomposite	Lyophilization	HPMC–PF 127	<100	[16]

^a^ Dried suspension formulation. HPMC: hydroxypropylmethyl cellulose, PF: Poloxamer (Pluronic), Tween: polysorbate. ^b^ If not reported directly, values were estimated based on the dissolution data. NR: not reported or dissolution was not performed under supersaturating (non-sink) conditions.

The main objective of this study is to test if HyNASDs can remarkably enhance the supersaturation of itraconazole (ITZ, a poorly soluble drug) nanoparticles during dissolution tests and assess the impact of various polymers, absence/presence of a surfactant, and drug particle size. Unlike previous studies with a fast crystallizer [31,33], HyNASDs were produced here with a slow crystallizer (ITZ) [32]. To this end, wet media milled aq. ITZ suspensions and ITZ solutions containing a polymer (HPC: hydroxypropyl cellulose, Sol: Soluplus, or VA64: Kollidon-VA64) were spray-dried to prepare ITZ HyNASDs and ITZ ASDs, respectively. Aqueous ITZ suspensions with 0.125% surfactant (SDS: sodium dodecyl sulfate) and without SDS were also prepared to elucidate the impact of SDS during in vitro drug release from the HyNASDs. The particle size distribution of the nanosuspensions and the spray-dried powders was characterized via laser diffraction. The solid-state characterization of the spray-dried powders was performed by X-ray powder diffraction (XRPD) and differential scanning calorimetry (DSC). The modified Washburn method was used to evaluate ITZ wettability enhancement by the HPC/Sol/VA64 with or without SDS. The release of ITZ from the spray-dried powders was studied using a USP II apparatus coupled with UV spectroscopy. Polarized light microscopy (PLM) was used to visually examine the physical change in the ASD particles upon contact with the dissolution buffer. To confirm that ITZ is a slow crystallizer, ITZ recrystallization behavior in the presence/absence of HPC/Sol/VA64 and SDS was examined via solvent-shift experiments. This study has demonstrated the remarkable capability of HyNASDs in boosting the drug supersaturation with nanoparticles of a slow crystallizer (ITZ). It has also established the primary importance of drug particle size and the polymer type as well as the secondary importance of the surfactant.

## 2. Materials and Methods

### 2.1. Materials

Itraconazole, ITZ (Jai Radhe Sales, Ahmedabad, India), was used as a model slow crystallizer [32] and a poorly soluble drug. The solubility of pure ITZ in deionized water and 0.1 N HCl solution at 37 °C is ~0.002 µg/mL and ~5 µg/mL [35], respectively. Its melting point temperature *T*_m_ and glass transition temperature *T*_g_ are 172 °C and 59 °C [34], respectively. SSL grade of Hydroxypropyl cellulose, HPC (Nisso America Inc., New York, NY, USA), has been widely used as a matrix polymer in drug nanocomposites [2]. Soluplus, Sol (BASF, Florham Park, NJ, USA), is a graft copolymer made of polyvinyl caprolactam–polyvinyl acetate–polyethylene glycol. Kollidon VA64 (BASF, Florham Park, NJ, USA) is a copolymer of vinylpyrrolidone and vinyl acetate. Sodium dodecyl sulfate, SDS (GFS Chemicals, Inc., Columbus, OH, USA), is an anionic surfactant, and was used as a wetting agent. Dichloromethane, DCM ACS reagent, ≥99.5% purity (VWR Chemicals BDH, Radnor, PA, USA), was used as the solvent to prepare drug–polymer solutions. Dimethyl sulfoxide, DMSO ACS reagent ≥99.9% purity (Fisher Scientific, Fair Lawn, NJ, USA), was used as the solvent to dissolve ITZ in the solvent-shift tests. Wear-resistant yttrium zirconia beads, Zirmil Y (Saint Gobain ZirPro, Mountainside, NJ, USA) with a median size of 430 µm were used as the milling media.

### 2.2. Preparation Methods

#### 2.2.1. Preparation of Feeds for Spray Drying Process

Aqueous suspension-based (W:water) feeds of ITZ prepared by wet media milling and organic solution-based (S:solvent) feeds of ITZ were fed to the spray dryer for the preparation of drug HyNASDs and ASDs, respectively. Table 2 presents the formulations of various 2.5% ITZ suspensions/solutions with HPC/Sol/VA64 in 240 mL deionized water/DCM. All the concentrations were calculated with respect to the total volume of the solvent (deionized water/DCM). Except for Sol, which will be shown to provide the highest supersaturation among all polymers, the drug:polymer mass ratio was fixed at 1:5 for both suspension-based (W) and solution-based (S) feeds for direct comparison. The impact of polymer loading on ITZ release during dissolution was studied by varying the ITZ:Sol ratio from 5:1 to 1:5 for the suspension-based (W) feeds. To elucidate the role of Sol and particle size of ITZ, an aq. suspension of as-received (micronized) ITZ with 1:5 ITZ:Sol (W-AR-Sol-1:5) was also prepared. Finally, to investigate the impact of SDS in the stabilization of the milled ITZ suspensions and ITZ release during dissolution tests, suspensions with the same drug:polymer mass ratios with SDS were also prepared. The SDS concentration was kept constant below the critical micelle concentration (CMC, 0.24% *w*/*v*) at 0.125% (*w*/*v*) to minimize potential Ostwald ripening.

In each milling experiment, a shear mixer with Catalog No. 14-503 (Fisher Scientific, Pittsburgh, PA, USA) was used to disperse as-received ITZ particles in the aq. stabilizer solutions. The resultant ITZ pre-suspensions were transferred to the holding tank of a Microcer wet stirred media mill (WSMM) (Netzsch Fine Particle Technology, LLC, Exton, PA, USA) with an 80 mL chamber. Milling conditions were guided by our prior work [36]. In addition, 196 g zirconia beads were placed in the milling chamber, while a screen with a 200 µm opening was used at the outlet of the milling chamber to retain the beads. Using a peristaltic pump, the suspension was recirculated through the chamber at a rate of 126 mL/min, and the suspension was milled for 65 min at a rotor speed of 4000 rpm. The temperature of the milling chamber was maintained below 34 °C throughout the milling with the help of a chiller (Advantage Engineering, Greenwood, IN, USA). A portion of each suspension was separated in a vial and stored for 7 days at 8 °C to assess the short-term physical stability. Also, the milled suspensions were refrigerated at 8 °C overnight before spray drying.

As a precursor for ASD production, 2.5% (*w*/*v*) ITZ solutions in DCM were prepared with three polymers (HPC, Sol, and VA64), while keeping the drug:polymer mass ratio constant at 1:5. Due to processability issues and unavailability of an appropriate solvent, solution-based (S) feeds with SDS were not prepared. After dissolving the drug–polymer into DCM using a magnetic stirrer, the solutions were sonicated for 30 min to ensure complete solubilization of the solids before feeding to the spray dryer.

#### 2.2.2. Production of Spray-Dried Powders

A Procept 4M8-Trix spray dryer (Zelzate, Belgium) was used to dry 200 g ITZ suspension/solution that was fed at 2.0 g/min. Drying air at 120 °C was fed at the rate of 0.32–0.35 m^3^/min for suspension-based (W) feeds, whereas drying air at 65 °C was fed at the rate of 0.27–0.30 m^3^/min for solution-based (S) feeds. These parameters were set based on the boiling point and evaporation enthalpy of the respective solvents. As water is much less volatile than DCM, a higher temperature and air flow rate were used to dry W feeds. The suspensions/solutions were atomized through a bi-fluid nozzle with 0.6 mm tip diameter at an atomization air pressure of 2 bar. A cyclone was used to separate the dried particles from the outlet air stream into a glass jar. The pressure difference was maintained at 54–62 mbar across the cyclone. After spray drying, a vacuum-desiccator was used to store dried particles in double plastic bags at room temperature before their characterization.

#### 2.2.3. Particle Sizing and Solid-State Characterization

Laser diffraction (LS 13 320, Beckman Coulter, Miami, FL, USA) based on Mie scattering theory was used to measure the drug particle size distribution (PSD) in the suspensions following the procedure described in [37]. Particle sizes were measured right after 65 min milling and 7-day storage at 8 °C in a refrigerator. During the measurements, the intensity was maintained at 40–50% while the obscuration was below 8.0%. Refractive index values are 1.68 for ITZ (drug) and 1.33 for deionized water (medium). A 2.0 mL milled suspension sample was diluted with 5.0 mL of the respective stabilizer solution and mixed using a vortex mixer with Model No: 945415 (Fisher Scientific, Pittsburgh, PA, USA) at 1500 rpm for 1 min before each measurement. A Rodos/Helos laser diffraction system (Sympatec, NJ, USA) was used to measure the PSD of the as-received ITZ and spray-dried powders, based on Fraunhofer theory, following the method in [21].

XRPD (PANanalytical, Westborough, MA, USA), equipped with Cu K_α_ radiation (λ = 1.5406 Å), was used to investigate the crystalline nature of the as-received ITZ, polymers (HPC/Sol/VA64), SDS, spray-dried samples, and physical mixtures (PMs). The PMs have the same formulations as those presented in Table 2, but were prepared via blending. All samples were scanned within the range of 5° to 40° at a rate of 0.165 s^−1^ in 2*θ* scanning mode. To estimate the %crystallinity of the spray-dried powders, the total area under four distinct, non-overlapping peaks of ITZ at characteristic diffraction angles of 17.5°, 17.9°, 20.3°, and 23.4° was calculated for both the PMs and the spray-dried powders using the equipment’s HighScore Plus software version 5.1and following the method in [33].

A Mettler-Toledo polymer analyzer (PolyDSC, Columbus, OH, USA) was used to perform DSC on the as-received ITZ, HPC, Sol, VA64, spray-dried samples, and physical mixtures (PMs). About 6–7 mg of each sample was used for the tests. The DSC data were analyzed using the integrated software (STARe 10) of the equipment. As-received ITZ and PMs were heated from 25 °C to 200 °C at a rate of 10 °C/min under nitrogen gas flow. Spray-dried samples were heated from 25 °C to 65 °C and held at 65 °C for 2 min to remove any residual solvent, then cooled back to 25 °C. In the last step, the samples were heated again at a rate of 10 °C/min from 25 °C to 200 °C. To assess the residual moisture/solvent content in the spray-dried samples, thermogravimetric analysis (TGA) was performed using a TGA/DSC1/SF Stare system (Mettler Toledo, Inc., Columbus, OH, USA). Each sample weighing ~6.0–7.0 mg was placed in a ceramic crucible and heated at a rate of 10 °C/min under nitrogen flow from 25 °C to 150 °C.

#### 2.2.4. Assessment of Actual Drug Content and Drug Release

To evaluate the actual drug content in the spray-dried powders, an assay test was conducted following the method in [36]. In this test, 100 mg of the spray-dried samples was dissolved in 20 mL of DCM under sonication for 40 min and stored overnight. Using a UV spectrophotometer (Agilent, Santa Clara, CA, USA) at a 260 nm wavelength, the absorbance of the samples was measured, while a pre-established calibration curve was used to calculate the drug concentration. For each formulation, mean drug content and the relative standard deviation (RSD) were calculated (*n* = 6). A Distek 2100C USP II (paddle apparatus) dissolution tester (North Brunswick, NJ, USA) was used to determine ITZ release from the as-received ITZ, spray-dried powders, and the physical mixtures (PMs) with 100 mg equivalent ITZ in 1000 mL 0.1 N HCl solution at 37 °C stirred with a paddle speed of 50 rpm. Considering that the solubility of ITZ in 0.1 N HCl is ~5.0 µg/mL at 37 °C, a high dose (100 mg) of ITZ would allow for supersaturating dissolution conditions. After pouring the powders into the dissolution medium, 4 mL samples were taken out manually at different time intervals up to 210 min. These aliquots were filtered with a 0.1 µm PVDF membrane-type syringe filter before UV spectroscopy measurements to minimize any confounding effect of the undissolved coarse drug aggregates [21]. The filtered samples were diluted with 0.1 N HCl solution kept at 37 °C before UV measurement. The concentration of ITZ was measured by UV spectroscopy at a 255 nm wavelength and calculated using a pre-established calibration curve. Six samples were tested for each formulation. Note that the wavelength during the assay test and the dissolution test was different due to different solvents used. In assay tests, the liquid medium was DCM, whereas the medium was 0.1 N HCl in the dissolution tests. As the PMs were not expected to provide much supersaturation, except for Sol-based PM of which the full dissolution profile over 210 min was measured, the supersaturation was only measured and reported at 210 min for PMs of the other polymers.

#### 2.2.5. ITZ Wettability Enhancement by Polymer Solutions with or without SDS

Using the modified Washburn method, the wettability of ITZ was analyzed. The experimental method was adapted from [28], and the details are provided in Section S1 of the Supplementary Material. The penetration rate of aqueous polymer solutions into a packed bed of as-received ITZ powder inside a cylindrical column was analyzed. The mass of liquid that penetrated the as-received ITZ powder bed was measured as a function of time using an Attension Sigma 700 (Biolin Scientific, Linthicum, MD, USA). Here, the liquids refer to the solutions of 12.5% polymer (HPC/Sol/VA64) with or w/o SDS, saturated with ITZ in 0.1 N HCl solution. Surface tension was measured using Attension Sigma 700 with a Wilhelmy plate, and the apparent shear viscosity was measured with an R/S Plus Rheometer (Brookfield Engineering, Middleboro, MA, USA). A wetting effectiveness factor, i.e., cos *θ*_ss_/cos *θ*_w,_ was calculated using the modified Washburn equation. Here, *θ*_ss_ is the contact angle between ITZ particles and the aq. HCl–stabilizer solutions, whereas *θ*_w_ is the contact angle between ITZ particles and the HCl solution. This ratio provides a rough measure of the drug wettability enhancement upon use of various stabilizers in the acidic medium. The wetting effectiveness factor gauges the extent to which the wettability of a hydrophobic drug is enhanced when polymers and/or surfactants are used as solutes in the wetting fluid.

#### 2.2.6. Precipitation Behavior of ITZ and Potential Impact of Polymers and SDS

A solvent-shift method was used to examine the impact of HPC/Sol/VA64 with or without SDS on the possible depletion of ITZ supersaturation (desupersaturation) from a pre-supersaturated ITZ solution, which will help to elucidate any ITZ crystallization in the dissolution tests. To generate supersaturation, a concentrated solution of ITZ (100 mg ITZ dissolved in 20 mL DMSO) was mixed with a pre-dissolved HPC/Sol/VA64 solution in 0.1 N HCl in the dissolution tester with a polymer concentration of 500 µg/mL, emulating a 1:5 ITZ:polymer ratio in the powders, with 0.0005% SDS or without SDS. The solvent-shift during the mixing generated a supersaturated solution of ITZ with a target concentration of 100 µg/mL initially, which corresponds to the complete dissolution of a 100 mg drug. Measuring the ITZ concentration over 210 min allowed us to determine when/if supersaturation was depleted due to ITZ crystallization. The other experimental conditions and concentration measurement methods were identical to those in the dissolution test. The experiments were performed in triplicate.

The visualization of possible drug recrystallization during dissolution due to imbibition of the buffer solution into an ASD compact was performed by adapting the procedure from earlier studies [28,33]. A loose compact of the spray-dried samples produced from solution-based feeds (feeds S1–S3) was mounted onto a glass slide. After the addition of ~30 µL of buffer solution (0.1 N HCl), the sample was observed under a polarized light microscope (PLM). Images were taken at 0, 1, 5, and 10 min from the moment of adding the acidic solution.

## 3. Results

### 3.1. Properties of ITZ Nanosuspensions Prepared via Wet Stirred Media Milling

Eight different ITZ suspensions (W2–W9) with HPC/Sol/VA64–0.125% SDS and w/o SDS were wet media milled. Table 3 shows the characteristic particle sizes of the suspensions after 65 min milling and after their 7-day storage. After milling for 65 min, *d*_50_ and *d*_90_ were in the range of 0.16–0.44 µm and 0.25–2.2 µm, respectively, for all formulations. As-received ITZ (unmilled) particles had *d*_10_: 2.05 ± 0.1 µm, *d*_50_: 8.82 ± 0.1 µm, and *d*_90_: 24.4 ± 0.2 µm. Therefore, it is concluded that wet media milling caused extensive size reduction in the large ITZ particles into nanoparticles; however, some suspensions exhibited aggregation during milling and/or storage. Two counteracting mechanisms operate simultaneously during wet media milling: breakage of the drug particles, fragments of already broken particles as well as aggregates (deaggregation) and aggregation of the particles [38,39]. The PSD and characteristic particle sizes like *d*_50_ and *d*_90_ vary in accordance with the competition between the rates of these two opposing mechanisms, i.e., breakage and aggregation. Also, nanoparticles can aggregate during storage due to Brownian motion and ensue collisions unless properly stabilized. Drug nanoparticles can form micron-sized aggregates in aqueous suspensions [5,6], which was also observed in the ITZ suspensions without SDS (except, W-HPC-1:5). Aggregation of ITZ particles was notable during the milling with Sol/VA64 without SDS (*d*_90_ > 1 μm in Table 3). Therefore, the effectiveness of the polymers for the stabilization of milled ITZ suspensions can be rank ordered as HPC > Sol > VA64 in the absence of SDS, which may be explained by the different extent of their adsorption onto the ITZ particles.

The aggregation was largely mitigated or slowed down owing to the use of 0.125% SDS along with the polymers. HPC/Sol–SDS was reported to have a synergistic stabilizing effect on multiple BCS Class II drug nanosuspensions during milling and storage [31,37]. Neutral polymers (HPC/Sol/VA64) imparted steric stabilization by being adsorbed on the surfaces of the drug nanoparticles [20,36], while the anionic surfactant (SDS) enhanced ITZ wettability/deaggregation and helped to stabilize the ITZ nanosuspensions via electrostatic repulsion. As will be discussed in Section 3.4.3, polymers alone and polymer–SDS combinations both reduced the surface tension and enhanced the ITZ wettability in 0.1 N HCl solution. As indicated by the higher wetting effectiveness factor, HPC rendered ITZ more wettable than VA64/Sol, especially when combined with SDS. The wettability is important to the deaggregation of the aggregates formed during milling, which allows full exposure of ITZ particle surfaces for polymer adsorption. Besides the different adsorption extents of the polymers on the ITZ particle surfaces, the lower wettability of ITZ by Sol as compared with HPC could be the reason for the large aggregates in Sol-based suspensions. On the other hand, with SDS, finer suspensions were obtained with Sol than with HPC, which suggests different interactions between the polymer–SDS.

### 3.2. Residual Moisture, Drug Content, and Particle Sizes of the Spray-Dried Powders

Despite the relatively short residence time in the spray dryer, the powders produced from both suspension-based (W) and solution-based (S) feeds were sufficiently dried, as indicated by TGA, which showed a weight loss of ~2.0% for the samples. The extremely large surface area generated by the atomization of the feed coupled with the high convective heat–mass transfer at a high air temperature enabled fast drying of the droplets in the drying chamber. The mean (actual) drug content after spray drying was close to the theoretical drug content, and all RSD values were below 6%: 0.49–5.70%, which signifies pharmaceutically acceptable content uniformity (see Table 4). Table 4 also shows that due to the higher total solid loading (more viscous feed) [22,36], coarser particles were produced when the polymer concentration was increased in the case of W2–W4. Coarser particles were produced for formulations with different polymers in the order HPC > Sol > VA64, which are in line with the aq. viscosity of the respective polymers in the suspensions, which are presented in Section 3.4.3. Unlike this clear and strong effect of the polymer, the effect of SDS did not show a clear pattern.

### 3.3. Solid State Characterization of the Spray-Dried Powders

**Figure 1:**
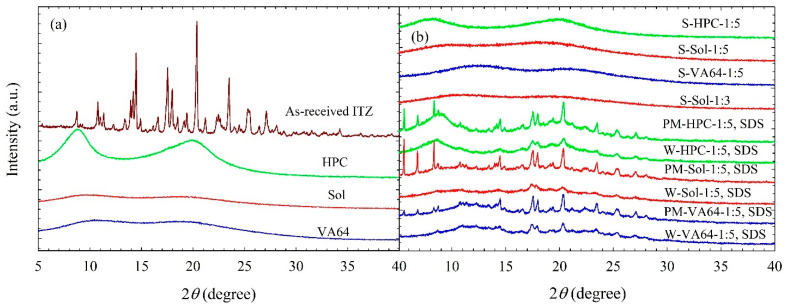
X-ray diffractograms of (**a**) as-received ITZ, HPC, Sol, and VA64; (**b**) spray-dried powders prepared using the ITZ solution-based (S) feeds without SDS, spray-dried powders prepared using ITZ suspension-based (W) feeds and their corresponding physical mixtures (PMs) with SDS.

**Figure 2:**
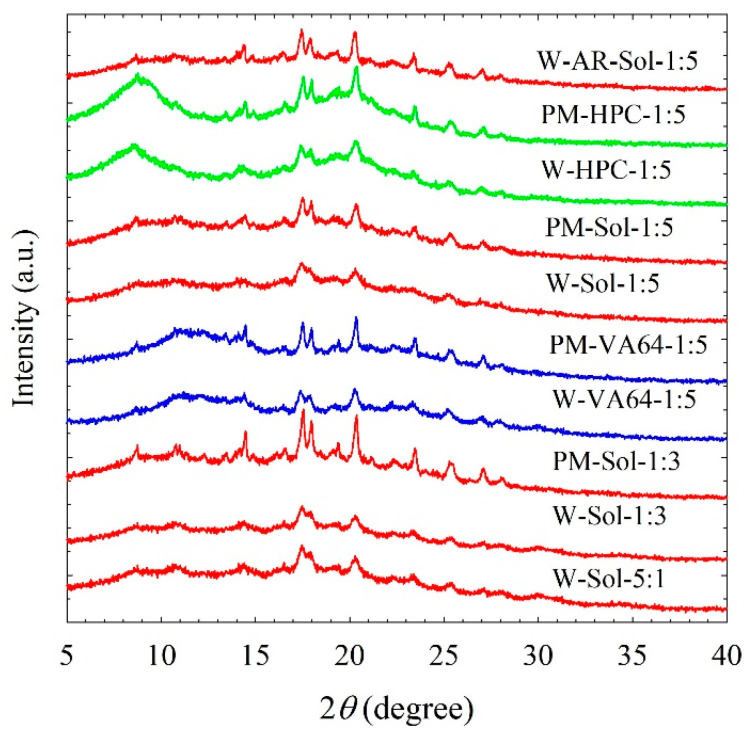
X-ray diffractograms of physical mixtures (PMs) of ITZ–HPC/Sol/VA64 and the spray-dried powders prepared using the ITZ suspension-based (W) feeds without SDS. The ratios represent the drug:polymer mass ratio. AR stands for the spray-dried powder prepared using a suspension-based feed of as-received (micronized) ITZ.

Figure 1 and Figure 2 illustrate the XRPD diffractograms of the as-received ITZ, HPC, Sol, VA64, physical mixtures (PMs), and of the spray-dried powders. X-ray diffractograms (Figure 1a) depict that as-received ITZ exhibited intense peak characteristics of a crystalline material, whereas HPC/Sol/VA64 exhibited halo patterns indicating an amorphous state. The physical mixtures (PMs) exhibited peaks at the same diffraction angles as those of the as-received ITZ, albeit with reduced intensity, which can be attributed to the dilution and surface coverage of ITZ microparticles with HPC/Sol/VA64 (Figure 1b). The peaks observed between 5° and 10° in the PMs correspond to the main characteristic peaks of crystalline SDS (Figure 1b). The main characteristic peaks of SDS are at 5.6°, 6.8°, and 8.3°. The SDS diffractogram was excluded from Figure 1 for proper scaling of ITZ peaks; readers are referred to Section S2 and Figure S2 of the Supplementary Materials for the diffractogram of SDS.

The diffractograms of the spray-dried powders produced from suspension-based (W) feeds with SDS (Figure 1b) and without SDS (Figure 2) did not remarkably differ, except for the peak intensities; they exhibited a similar pattern to those of the PMs. This finding suggests that spray drying of the milled suspensions led to the formation of powders that were largely crystalline in nature. The characteristics peaks of SDS present in the PMs were absent in the diffractograms of the spray-dried powders as a small amount of SDS was most likely to be molecularly dispersed in the polymeric matrix of the spray-dried powders. Interestingly, the diffractograms of the spray-dried powders show reduced peak intensities as compared with their respective PMs, beyond the dilution effect of the polymers. Wet milling followed by spray drying led to a reduction in crystallinity and the formation of notable amorphous (mostly >10%) ITZ (Table 5). In the presence of high polymer loading in the suspensions mentioned here, amorphization of ITZ took place during milling and spray drying. Looking at the XRPD diffractograms (Figure 1b and Figure 2), the reduction in peak intensity was more pronounced in the case of Sol, confirming more amorphous ITZ formation in the Sol formulations than in the HPC and VA64 formulations.

These findings imply that (i) amorphous ITZ formed due to ITZ–polymer molecular interactions and/or solubilization of the surface layer of nanoparticles by the polymer during the spray drying and (ii) Sol appears to favor the amorphization of ITZ more than HPC and VA64. It is likely that the presence of ITZ nanoparticles and their aggregates with a large surface area and higher polymer loading (more ITZ–polymer interactions and higher ITZ solubilization in the polymer) could have favored the formation of amorphous ITZ. Based on the drying of a drug nanosuspension in [40], it is proposed that the polymeric matrix of the spray-dried particles encapsulated drug nanocrystals/aggregates, surrounded by a layer of the amorphous drug molecularly dispersed in the polymer. In other words, the XRPD diagrams (Figure 1b and Figure 2) and %crystallinity (Table 5) confirm the formation of ITZ-HyNASDs upon the drying of ITZ nanosuspensions. On the other hand, XRPD diffractograms (Figure 1b) of the spray-dried powders produced from solution-based (S) feeds showed a halo pattern instead of any characteristic diffraction peaks of ITZ. These halo patterns confirm that ITZ was dispersed molecularly into the polymer matrices, forming amorphous solid dispersions (ASDs).

Generally, the solubility parameter difference between a drug and polymer may be used to assess their theoretical miscibility. They are classified as miscible, partially miscible, and immiscible for the differences below 7.0 MPa^1/2^, between 7.0 MPa^1/2^ and 10 MPa^1/2^, and above 10 MPa^1/2^, respectively [41,42]. The solubility parameters of ITZ, HPC, Sol, and VA64 are 22.6 [43], 24.0 [44], 19.4, and 19.7 [43] MPa^1/2^, respectively. The solubility parameter differences between ITZ–Sol and ITZ–VA64, and ITZ–HPC are 3.2, 2.9, and 1.4 MPa^1/2^, respectively, which suggests that ITZ–Sol/VA64/HPC are all miscible based on the solubility parameter difference and very likely to produce ASDs of ITZ. While being useful, the theoretical models behind the solubility parameter predictions have various limitations [32], and hence should be used with caution as rough estimates of drug–polymer miscibility. As will be discussed further below, the DSC thermograms in Section S3 of the Supplementary Materials show that the physical mixtures lowered the melting point temperature of ITZ due to the miscibility of the polymer with ITZ, and this depression was rank ordered Sol ≈ VA 64 > HPC. The detection of melting point and non-zero fusion enthalpy in the thermogram of ITZ–HPC ASD (see S-HPC-1:5 Table 5) could be indicative of the lower extent of miscibility and formation of drug-rich domains in this ASD.

**Figure 3:**
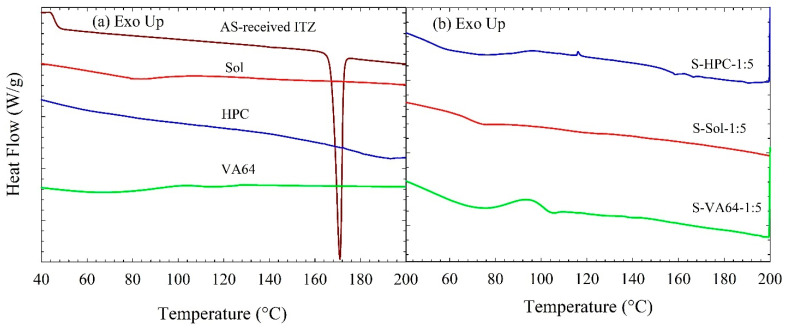
DSC thermograms of (**a**) as-received ITZ, HPC, Sol, and VA64; (**b**) spray-dried powders prepared using the ITZ solution-based (S) feeds in the formulation.

The DSC thermograms in Figure 3a show an endothermic peak associated with the melting of as-received ITZ, with a melting point temperature *T*_m_ of 171 °C and a fusion enthalpy Δ*H*_f_ of 70.9 J/g; a glass transition for Sol at 72.4 °C, a glass transition for VA64 at 102 °C, and a slight endothermic event around 170–200 °C for HPC likely due to the melting of the small crystalline domains of largely amorphous HPC (crystallinity was undetectable by XRPD) [45]. Due to the limitation of the equipment, *T*_g_ of HPC could not be measured, but it was reported to be 81.8 °C [46]. The spray drying of the solvent-based feeds with Sol and VA64 did not exhibit any fusion event in their thermograms (Figure 3b), whereas that with HPC exhibited a small endothermic (fusion) event. Indeed, for the S-HPC formulation, a small recrystallization event at 116 °C followed by a melting event at 158 °C were observed, which might be due to the phase separation of amorphous ITZ and HPC during heating. Due to stronger molecular interactions and good miscibility, a recrystallization event at high temperature was not observed for ITZ–Sol and ITZ–VA64 formulations during the DSC scan. For the spray-dried powders prepared from the drug–polymer solutions (Figure 3b), a single *T*_g_ was observed for all the formulations, confirming the formation of molecular-level dispersion [47] (see Table 5). Overall, the DSC thermograms and XRPD diffractograms confirm the formation of ASDs when solution-based feeds were spray-dried.

**Figure 4:**
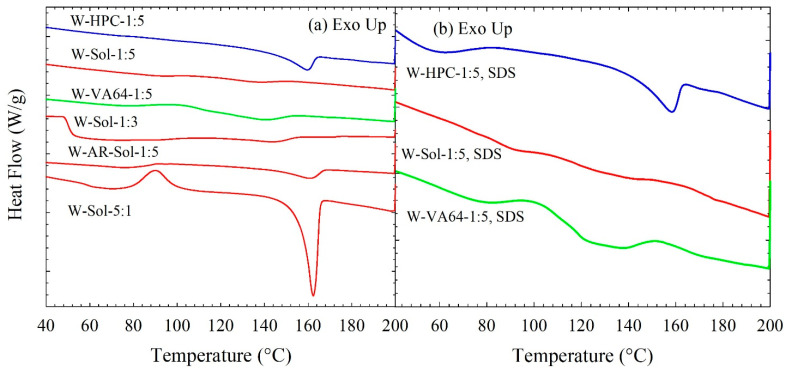
DSC thermograms of spray-dried powders prepared using the ITZ suspension-based (W) feeds: (**a**) without SDS in the formulation and (**b**) with SDS in the formulation. AR stands for the spray-dried powder prepared using a suspension-based feed of as-received (micronized) ITZ.

The DSC thermograms in Figure 4 and associated data in Table 5 suggest that spray drying of ITZ nanosuspensions led to a drastic melting point depression (high Δ*T*_m_), up to 41 °C, and reduction in Δ*H*_f_ even if the values were corrected for dilution with polymers and reduced crystallinity (see Figure S3 and Table S1). The significant melting point depression in the drug–polymer mixtures is an indicator of drug–polymer miscibility [48]. In general, higher polymer loading (lower ITZ:polymer mass ratio) led to lower *T_m_* as compared with the as-received ITZ crystals, higher Δ*T*_m_, and lower Δ*H*_f_, regardless of the presence/absence of SDS, which implies significant ITZ–polymer molecular interactions. This finding is also supported by the DSC thermogram of the spray-dried W2 formulation (see Figure 4a, where the lowest polymer concentration was also shown). The *T*_m_ = 162 °C of W2 was closer to that of the as-received ITZ, and W2 had the highest Δ*H*_f_ (34.4 J/g) among all the spray-dried powders. Without exception, with identical polymer/SDS compositions, the spray-dried powders with Sol had higher Δ*T*_m_ and lower Δ*H*_f_ than those with HPC and VA64, which could be explained by (i) stronger ITZ–Sol interactions, (ii) higher initial amorphous content in the Sol-based spray-dried powders, and (iii) higher extent of solubilization of ITZ in the polymer melt at high temperatures due to the thermal treatment during the DSC scan. Compared with the clear trends regarding the impact of different polymers for formulations with/without SDS, the trends for SDS impact were not as strong and as clear.

Overall, the XRPD and DSC results suggest that spray drying of ITZ–polymer nanosuspensions with/without SDS led to the formation of drug HyNASDs and that of ITZ–polymer solutions led to the formation of ASDs.

### 3.4. ITZ Release from the Spray-Dried Powders

#### 3.4.1. Spray-Dried Powders vs. as-Received Drug and Physical Mixtures

The temporal evolution of ITZ release from the spray-dried powders and the PMs with 1:5 ITZ:Sol containing a 100 mg equivalent ITZ dose in 1000 mL 0.1 N HCl solution at 37 °C was investigated. The bulk dissolution medium demonstrated supersaturation for this high drug dose as the thermodynamic solubility of ITZ was ~5.0 µg/mL at the specific dissolution conditions [35]. Unless otherwise specified, all supersaturation values are relative to thermodynamic solubility of ITZ in 0.1 N HCl and calculated at 210 min.

**Figure 5:**
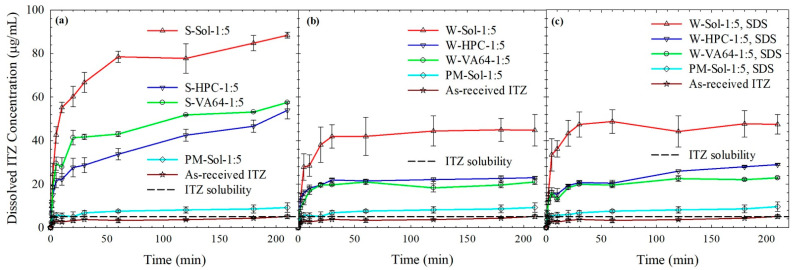
Evolution of drug release in 0.1 N aq. HCl medium from as-received ITZ, physical mixtures (PMs) with 1:5 ITZ:Sol mass ratio with and without SDS, and spray-dried powders prepared from: (**a**) ITZ solution-based (S) feeds, (**b**) ITZ suspension-based (W) feeds without SDS, and (**c**) ITZ suspension-based (W) feeds with SDS. Dissolution sample size is equivalent to 100 mg ITZ (*n = 6*).

Figure 5 shows that the mere presence of Sol (see 1:5 ITZ:Sol mass ratio PM formulations, with or without SDS) could increase the ITZ release rate without any prior processing, i.e., wet-milling–spray drying of the as-received (micronized) ITZ particles. In addition, 90% and 80% higher apparent solubility of ITZ occurred in ITZ–Sol PMs with and without SDS, respectively. For PM-HPC-1:5, PM-VA64-1:5, PM-VA64-1:5, and the same PMs with SDS, the apparent solubility increases were all within 60–70% (only measured at 210 min, the profiles were not established due to low supersaturation). This relatively low supersaturation from the PMs could be explained by the higher solubility of ITZ in the dissolution medium due to the presence of polymer/SDS in the formulation. More importantly, Figure 5 clearly depicts that all spray-dried powders released ITZ faster, generating higher supersaturation, than the PMs owing to the presence of amorphous ITZ, which has higher kinetic solubility than crystalline ITZ.

#### 3.4.2. ITZ Release from HyNASDs and ASDs

Considering that the major shortcoming of traditional drug nanocomposites with low polymer loadings as compared with amorphous solid dispersions (ASDs) is their limited supersaturation capability in dissolution media, the examination of drug dissolution under supersaturating conditions is critical. The most remarkable feature of Figure 5 is that Sol-based HyNASDs (nanocomposites with notable amorphous contents) attained 790% (Figure 5b) and 850% (Figure 5c) ITZ supersaturation without and with SDS, respectively. Such high supersaturation during a dissolution test has not been reported for drug nanocomposites before, except for ASDs. A cursory look at Figure 5 also reveals some general trends: (i) Sol-based formulations generated much higher supersaturation than HPC/VA64-based formulations for both HyNASDs and ASDs and (ii) the impact of the polymer was more notable than that of the absence/presence of the surfactant. Without SDS in the formulation, 360%, 320%, and 790% relative supersaturation values were attained by HPC, VA64, and Sol-based HyNASDs, respectively. On the other hand, in the presence of SDS, 480%, 360%, and 850% relative supersaturation values were achieved from HPC, VA64, and Sol-based HyNASDs, respectively. The SDS impact was the most obvious for the W-HPC-1:5 formulation. Overall, these results point to the criticality of the wet-media milling in preparing drug nanoparticles, wettability of the spray-dried powder, which was enhanced by polymer/SDS, ITZ–polymer miscibility, and ITZ–polymer interaction and solubilization of the ITZ by the polymers. Furthermore, 850% relative supersaturation from Sol–SDS-based formulation can be explained by the stronger ITZ–Sol interactions and micelle formation of the Sol–SDS combination, which is known to enhance the solubility of the drug [49].

A comparison of Figure 5a (ASDs) and Figure 5b (HyNASDs) indicates that ASDs outperformed HyNASDs, owing to the presence of 100% amorphous ITZ in the former. However, it is remarkable to see such high supersaturation (up to 790%) from the HyNASDs. Figure 5a shows that Sol-based ASDs exhibited higher supersaturation than HPC/VA64-based ASDs. In addition, 980%, 1050%, and 1670% relative supersaturation was attained by HPC, VA64, and Sol based formulations, respectively, at 210 min. The lack of any supramolecular structure in amorphous drugs, especially for a slowly crystallizing drug like ITZ, allows for high apparent solubility and fast drug supersaturation from ASDs. On the other hand, HyNASDs contain 70%–96% nanocrystals whose solubility islargely limited by the thermodynamic equilibrium solubility. Nanocrystals with sizes < 100 nm could exhibit higher saturation solubility due to their high curvature, as described by the Kelvin and the Ostwald–Freundlich equation [13]. As the majority of the drug particles in the HyNASDs are above 100 nm (refer to Table 3), we did not expect that such curvature effects could explain the significant supersaturation enhancement observed in HyNASDs. Moreover, there is much smaller amorphous content in the HyNASDs than in the ASDs; hence, it is not surprising that the ASDs outperformed the HyNASDs in terms of supersaturation generation. The solubility parameter differences suggest all polymers are miscible with ITZ, while the melting point depression implied in Table 5 suggests the miscibility of ITZ with the polymers is rank ordered as Sol > VA64 > HPC. The supersaturation generation in Figure 5a correlated positively with the ITZ–polymer miscibility inferred from DSC. The remarkably high ITZ supersaturation achieved by Sol-based ASDs as compared with HPC/VA64-based ASDs can be explained by the greater miscibility/interactions with ITZ and higher ITZ solubilization in Sol micelles in the dissolution medium. The continuously rising profiles during 210 min dissolution in Figure 5a suggests that there is no solution-mediated precipitation or recrystallization of ITZ during the dissolution. This aspect was separately investigated via independent solvent-shift tests in the next section.

#### 3.4.3. Insights from Wettability, Solvent-Shift, and Water-Imbibition Tests

During dissolution, as the dissolution medium is wetted and imbibes into the spray-dried particles, their polymer dissolves, and the particles redisperse into smaller ITZ–polymer/SDS clusters depending on the wettability, while their amorphous ITZ fraction contributes to the speed of the dissolution. In the polymer/SDS-rich microenvironment of the clusters, ITZ could be solubilized by the polymer/SDS, and the rate of this process depends on the cluster/particle size as well as ITZ particle size inside these clusters, and presence/absence of SDS. Depending on the polymer–drug miscibility and interactions, the amorphous content of the HyNASDs may phase-separate and recrystallize upon contact with the dissolution medium [50] because 0.1 N HCl solution acts as a plasticizing agent, reducing the glass transition temperature of the amorphous component of HyNASDs and enhancing the mobility of the drug molecules [50]. Finally, the supersaturated ITZ in the dissolution medium and the released drug nanoparticles form a metastable system. From a purely thermodynamic perspective, the chemical potential of solute in a supersaturated solution is higher than that of crystal, and it is in a high-energy metastable state, with a tendency to return to the equilibrium state with the lowest energy. The maintenance of high drug supersaturation is an important factor to improve membrane permeability. Hence, amorphous ITZ may recrystallize on existing ITZ nanoparticles of HyNASDs and cause their growth in time, which in turn can cause reduced supersaturation. On the other hand, strong drug–polymer interactions can reduce the molecular mobility, delay recrystallization onset time, and the extent of recrystallization in the dissolution medium [51]. Below, we will further analyze these aspects with additional experiments.

Let us examine the role of polymer–SDS on the wettability of the relatively hydrophobic drug. The time-wise evolution of the penetration of acidic solution–various acidic polymer solutions into a packed bed of ITZ powder is presented in Figure S1 of the Supplementary Material, and various properties of the liquids measured and the wetting enhancement factor cos *θ*_ss_/cos *θ*_w_ are presented in Table 6. The higher enhancement factor suggests greater wettability provided by the polymers in acidic solution to the hydrophobic drug. As expected, all three polymers are surface-active and reduced the surface tension when present in acidic solution, which is more pronounced in the presence of SDS. (Table 6). The relative wettability of the hydrophobic drug in acidic solution was improved by the polymer/polymer–SDS as follows: HPC > VA64 > Sol. Comparing Figure 5b with 5c, we note that the presence of SDS enhanced the extent of supersaturation (most notably for W-HPC-1:5) possibly due to the smaller drug particle size, greater wettability enhancement, along with the higher amorphous content compared to the formulations without SDS (refer to Table 3, Table 5 and Table 6).

Despite providing the lowest wettability enhancement, as compared with HPC/VA64, the Sol-based formulations attained the highest extent of supersaturation. Despite Sol’s relatively lower wetting effectiveness, its strong interaction with ITZ and micelle formation capability help to reach a higher extent of supersaturation compared to HPC/VA64. Sol is a bifunctional polymer and can act as both a polymeric carrier of solid dispersions and as a solubilizer through the formation of micelles in the aqueous medium [52]. The CMC of Sol in water at room temperature was reported to be 11.7 µg/mL [53]. Clearly, the Sol concentration in the dissolution medium (500 µg/mL) was way above the CMC of Sol, and the increased drug solubility could be partly ascribed to the micellar solubilization of ITZ by Sol. Thiry et al. [54] reported the micelle size of Sol in 0.1 N HCl to be 70 nm. Sol micelles can encapsulate the ITZ molecules in their hydrophobic core, which could partly explain the high ITZ supersaturation in the dissolution tests via Sol’s micellar solubilization mechanism.

**Figure 6:**
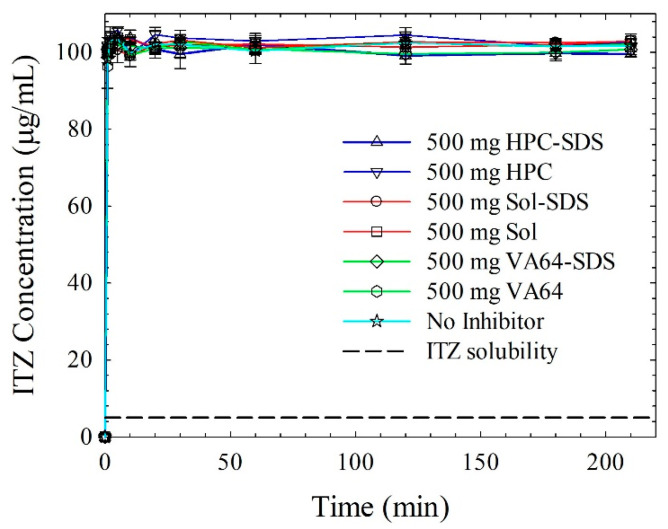
Evolution of ITZ concentration when 20 mL ITZ–DMSO solution was mixed with 1000 mL 0.1 N HCl solutions of 500 µg/mL of HPC/Sol/VA64–5 µg/mL SDS or without SDS (corresponding to 1:5 drug:polymer formulations). The 0.1 N aq. HCl solution has no recrystallization inhibitor. The initial concentration of ITZ right after mixing was targeted at 100 µg/mL (*n* = 3).

With the objective of gaining additional insights into the ITZ recrystallization behavior in the presence of Sol/HPC/VA64 with and without SDS in supersaturated ITZ solutions, we carried out solvent-shift tests. The solvent shifted from DMSO (high solubility of ITZ) to DMSO–0.1 N HCl mixture (low solubility of ITZ), thus creating supersaturation (see Figure 6). Peak ITZ supersaturation was quickly attained upon mixing the ITZ solution with the acidic solution, and it was maintained up to ~210 min with all the polymers along with and without SDS. This is not surprising because even in the absence of any inhibitor, ITZ did not precipitate within the experimental time, which is due to the intrinsically slow crystallizing nature of ITZ [32]. This finding independently explains the monotonic rising supersaturation during the dissolution of the ASDs (refer to Figure 5a) because there was no desupersaturation due to recrystallization of ITZ. The recrystallization behavior, the dissolution profiles, and the extent of supersaturation exhibited by ITZ were remarkably different from griseofulvin (GF), a fast crystallizer used in our previous study [31]. The slow intrinsic crystallization tendence of ITZ was the driver of the much higher supersaturation from ITZ HyNASDs than that from GF HyNASDs. On a separate note, the solvent-shift test in the absence of “nanocrystalline seeds” is incapable of explaining the potential desupersaturation due to the growth of residual ITZ nanocrystals in ASDs and nanocrystals in HyNASDS under supersaturation. Such detailed nanoseeded solvent-shift tests have been recently developed [45] and will be used in a future study.

**Figure 7:**
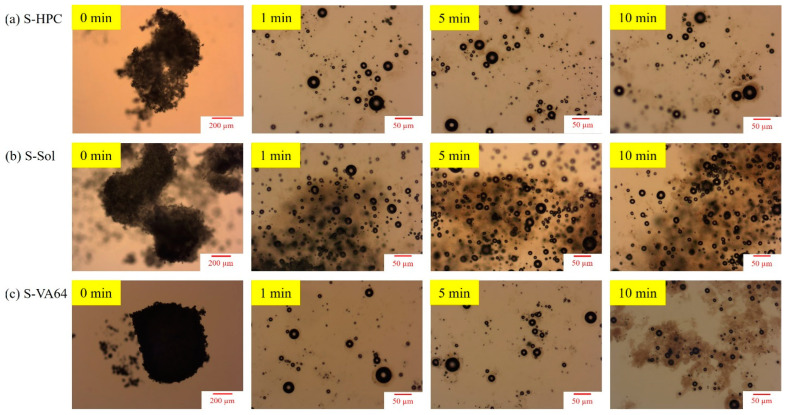
PLM images of a loose compact of the spray-dried ASD particles with 1:5 drug:polymer mass ratio in 30 µL 0.1 N HCl solution: (**a**) S-HPC, (**b**) S-Sol, and (**c**) S-VA64, respectively. The images were taken at 0 (before adding HCl solution), 1, 5, and 10 min after the addition of HCl solution. Except 0 min image (5× magnification, scale bar: 200 µm), which focused on the compact, all other images focused on particles that emanated from the surface, which were captured at 20× magnification (scale bar: 50 µm).

To assess if any phase separation and recrystallization of ITZ-ASDs occurred during dissolution, let us examine the PLM images (Figure 7) of loose ASD compacts imbibed with a 30 µL of 0.1 N HCl droplet. The addition of 0.1 N HCl droplets on the ASD compacts did not cause any recrystallization of amorphous ITZ regardless of the formulation (S1/S2/S3). Therefore, these images imply that a phase separation did not occur and the undissolved ITZ in the ASD particles stayed in the amorphous form, which facilitated the supersaturation generation of ITZ. This finding along with the supersaturation maintenance during the 210 min solvent-shift test (refer to Figure 6) corroborate that the ASDs generated by the spray drying did not phase-separate or undergo solution-mediated precipitation during the dissolution. In a previous study [55], GF–HPC and GF–VA64 ASD particles prepared by spray drying, when imbibed with deionized water, exhibited the formation of shiny crystals in the PLM images within 10 min of water imbibition.

#### 3.4.4. Dissolution of HyNASDs: Polymer Loading and ITZ Particle Size Effect

To gain insights into the polymer loading and ITZ particle size effects in drug release from ITZ-HyNASDs, additional dissolution experiments were carried out with different spray-dried powders prepared using suspension-based (W1–W3) feeds. To study the polymer loading effect, W-Sol-5:1 (W2), W-Sol-1:3 (W3), and W-Sol-1:5 (W4) suspensions were wet-milled followed by spray drying. ITZ nanoparticles with similar sizes were produced from both W-Sol-1:3 (*d*_50_ = 0.28 ± 0.01 µm) and W-Sol-1:5 (*d*_50_ = 0.26 ± 0.01 µm) suspensions, whereas slightly bigger ITZ nanoparticles were produced from W-Sol-5:1 (*d*_50_ = 0.36 ± 0.00 µm).

**Figure 8:**
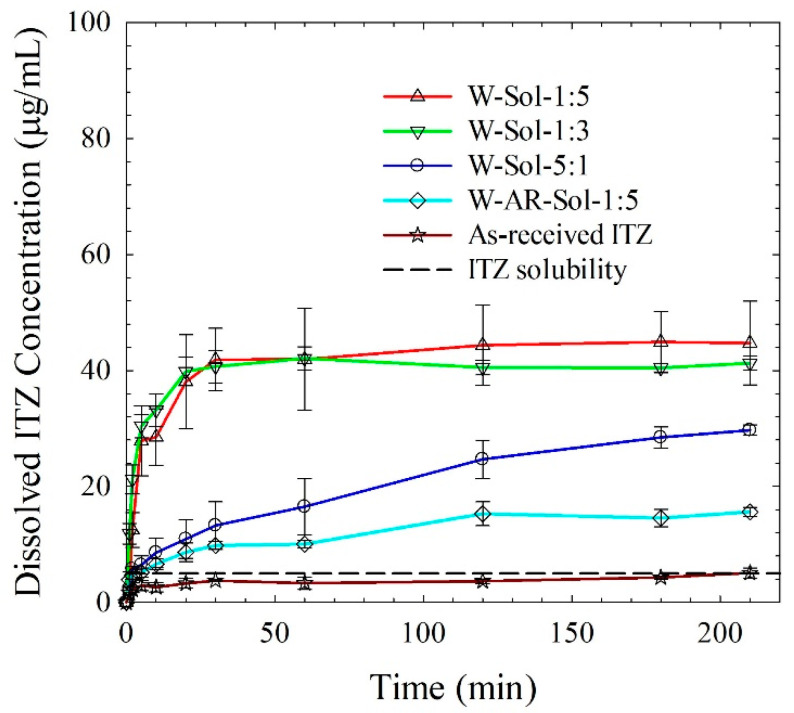
Evolution of ITZ release from as-received ITZ and spray-dried powders prepared from ITZ suspension-based (W) feeds with 5:1, 1:3 and 1:5 ITZ:Sol mass ratios. W-AR-Sol-1:5 refers to the spray-dried powder prepared using a suspension-based feed of as-received (micronized) ITZ. Dissolution sample size equivalent to 100 mg ITZ dose (*n* = 6).

The extent of supersaturation at 210 min was higher upon an increase in Sol loading: W-Sol-5:1 (490%), W-Sol-1:3 (730%), and W-Sol-1:5 (790%) (Figure 8). This might be attributed partly to the amorphous content in the respective spray-dried powders (HyNASDs): W-Sol-5:1 (16%) and W-Sol-1:3 (18%), and W-Sol-1:5 (20%) (refer to Table 5). More importantly, higher Sol loading provides more micellar solubilization and more interactions with ITZ particle surfaces. Both mechanisms contributed to the high supersaturation from HyNASDs having a high Sol loading. Therefore, there is a clear trend: an increase in Sol loading led to a higher amorphous content, higher micellar solubilization, and eventually higher ITZ supersaturation, while the precursor ITZ nanoparticle suspensions used to prepare these spray-dried powders did not have drastically different ITZ particle sizes.

It can be argued that the higher supersaturation in HyNASDs as compared to the traditional nanocomposites is solely due to ITZ–Sol interactions/miscibility and solubilization of ITZ by Sol micelles. W-AR-Sol-1:5 was prepared by spray drying the aqueous suspension of as-received (micronized, *d*_50_: 8.82 µm) ITZ microcrystals with Sol. After spray drying of AR-Sol-1:5, ~13% amorphous content was measured, which might be due to the interaction/miscibility of ITZ–Sol. W-Sol-1:5, which contains drug nanocrystals with *d*_50_: 0.26 µm and ~20% amorphous ITZ, generated four times more supersaturation than W-AR-Sol-1:5 (790% vs. 210%), demonstrating the importance of drug particle size in HyNASDs for supersaturation generation. Additionally, crystalline ITZ particles can be barely solubilized by Sol [56,57]; thus, micellar solubilization of crystalline ITZ by Sol cannot fully account for the observed high supersaturation. However, without any prior processing, achieving 210% is also quite impressive, which might be due to the amorphization of AR-Sol-1:5 (~13%) during spray drying through ITZ–Sol interactions and solubilization of ITZ in Sol micelles. The solubilization of ITZ particles (microparticles or nanoparticles) in the Sol matrix and supersaturation generation during the dissolution is a kinetically driven process, which is limited by the size of the particles: faster solubilization and higher supersaturation occurred when ITZ nanoparticles were encapsulated by the Sol matrix (HyNASD) as compared with the micronized ITZ particles owing to the approximately 40-times larger surface area of the nanoparticles.

### 3.5. Future Improvement Strategies for HyNASDs and Outlook

Considering that ASDs have storage stability challenges, HyNASDs may become competitive compared to ASDs for some drugs if they exhibit better storage stability. To this end, in a future study, the physical stability of the HyNASDs vs. ASDs will be examined under various temperature–relative humidity conditions. Moreover, HyNASDs’ performance can be further improved via formulation–process optimization, such as a change in the solvent, further reduction in the drug nanoparticle size via intensified wet stirred media milling [58], formulations with different types of polymers, binary polymers, and polymer–surfactant mixtures. Finally, significant supersaturation enhancement may not proportionally improve drug absorbance, and this aspect as well as the potential effect of SDS on membrane permeability of the drug warrants investigation via in vitro permeability tests, in vivo resorption assessments, or in vivo animal studies.

## 4. Conclusions

Spray drying of wet-milled ITZ suspensions with 1:5 ITZ:polymer (HPC/Sol/VA64) loading along with/without SDS led to the formation of a special class of nanocomposites, HyNASDs, which contain drug nanocrystals and aggregates surrounded by a notable amorphous drug (~5–30%). The most striking finding from this study is that despite containing >70% nanocrystals, HyNASDs demonstrated high values of supersaturation (up to ~800% within 210 min). On the other hand, ASD formulations generated higher supersaturation (up to 1670%) than HyNASDs. A higher miscibility and stronger interactions were indicated for ITZ–Sol than for ITZ–HPC/VA64. The high supersaturation generation capability of HyNASDs was explained by the slow crystallizing nature of ITZ in the dissolution medium, relatively high polymer loading with respect to ITZ, interactions/miscibility of ITZ–polymers as well as the size of the ITZ (nano)crystals in the polymeric matrix. Higher supersaturation from Sol-based HyNASDs compared to HPC/VA64-based HyNASDs was explained by the higher miscibility of Sol–ITZ and micellar solubilization capability of Sol. The presence of SDS enhanced supersaturation; but this effect was the most notable for the HPC-based HyNASD. Overall, HyNASDs could remarkably increase supersaturation from nanoparticle-based formulations of slowly crystallizing drugs, which may render them competitive to ASDs.

## Figures and Tables

**Table 2:** Formulations and compositions of the ITZ–HPC/Sol/VA64 suspensions (W) with or without SDS and solutions (S) without SDS fed to the spray dryer.

ID	Formulation ^a^	ITZ (% *w*/*v*) ^b^	SDS (% *w*/*v*) ^b^	Polymers (% *w*/*v*) ^b^			Water (mL)	DCM (mL)
				Sol	HPC	VA64		
W1	W-AR-Sol-1:5	2.5	0	12.5	-	-	240	-
W2	W-Sol-5:1	2.5	0	0.5	-	-	240	-
W3	W-Sol-1:3	2.5	0	7.5	-	-	240	-
W4	W-Sol-1:5	2.5	0	12.5	-	-	240	-
W5	W-HPC-1:5	2.5	0	-	12.5	-	240	-
W6	W-VA64-1:5	2.5	0	-	-	12.5	240	-
W7	W-Sol-1:5, SDS	2.5	0.125	12.5	-	-	240	-
W8	W-HPC-1:5, SDS	2.5	0.125	-	12.5	-	240	-
W9	W-VA64-1:5, SDS	2.5	0.125	-	-	12.5	240	-
S1	S-Sol-1:5	2.5	0	12.5	-	-	-	240
S2	S-HPC-1:5	2.5	0	-	12.5	-	-	240
S3	S-VA64-1:5	2.5	0	-	-	12.5	-	240

^a^ W denotes suspension-based feed; S denotes solution-based feed; AR denotes as-received. ^b^ % *w*/*v*, with respect to the total solvent volume (240 mL).

**Table 3:** Particle size statistics of the ITZ suspensions after 65 min milling and 7-day storage at 8 °C.

ID	Formulation ^a^	After 65 min Milling (µm)			After 7-Day Storage (µm)		
		*d*_10_ ± SD	*d*_50_ ± SD	*d*_90_ ± SD	*d*_10_ ± SD	*d*_50_ ± SD	*d*_90_ ± SD
W1	W-AR-Sol-1:5	—	—	—	—	—	—
W2	W-Sol-5:1	0.18 ± 0.00	0.36 ± 0.00	2.11 ± 0.02	0.19 ± 0.00	0.37 ± 0.04	2.14 ± 0.00
W3	W-Sol-1:3	0.15 ± 0.01	0.28 ± 0.01	1.68 ± 0.11	0.16 ± 0.00	0.26 ± 0.00	1.82 ± 0.01
W4	W-Sol-1:5	0.13 ± 0.00	0.26 ± 0.01	1.85 ± 0.08	0.14 ± 0.00	0.37 ± 0.00	2.16 ± 0.20
W5	W-HPC-1:5	0.13 ± 0.00	0.21 ± 0.00	0.32 ± 0.01	0.15 ± 0.01	0.23 ± 0.02	0.35 ± 0.02
W6	W-VA64-1:5	0.18 ± 0.00	0.44 ± 0.02	2.19 ± 0.01	0.18 ± 0.00	0.53 ± 0.06	2.18 ± 0.00
W7	W-Sol-1:5, SDS	0.11 ± 0.00	0.16 ± 0.00	0.25 ± 0.01	0.12 ± 0.00	0.18 ± 0.00	0.25 ± 0.01
W8	W-HPC-1:5, SDS	0.12 ± 0.00	0.18 ± 0.00	0.25 ± 0.01	0.12 ± 0.00	0.18 ± 0.00	0.25 ± 0.01
W9	W-VA64-1:5, SDS	0.14 ± 0.00	0.24 ± 0.01	0.45 ± 0.02	0.15 ± 0.00	0.27 ± 0.01	1.81 ± 0.12

^a^ 1:5, 1:3, and 5:1 drug:polymer mass ratios were used in the formulation; AR denotes as-received.

**Table 4:** Particle sizes of the spray-dried powders and their drug content.

ID	Formulation ^a^	Characteristic Particle Size (µm)			Theoretical Drug Content (% *w*/*w*) ^b^	Actual Drug Content, RSD, (% *w*/*w*, %)
		*d*_10_ ± SD	*d*_50_ ± SD	*d*_90_ ± SD		
W1	W-Sol-AR-1:5	5.41 ± 0.2	15.5 ± 0.2	31.9 ± 0.3	16.7	14.9, 1.01
W2	W-Sol-5:1	3.82 ± 0.1	8.21 ± 0.2	18.4 ± 0.4	83.3	74.7, 2.41
W3	W-Sol-1:3	5.53 ± 0.1	17.4 ± 0.2	39.7 ± 0.6	25.0	25.6, 1.06
W4	W-Sol-1:5	7.32 ± 0.4	21.8 ± 0.3	47.6 ± 0.3	16.7	17.1, 0.49
W5	W-HPC-1:5	7.81 ± 0.3	23.1 ± 0.1	54.7 ± 1.9	16.7	17.2, 3.56
W6	W-VA64-1:5	2.74 ± 0.6	9.98 ± 0.5	24.0 ± 0.9	16.7	17.1, 4.31
W7	W-Sol-1:5, SDS	4.95 ± 0.2	13.5 ± 0.4	27.9 ± 0.2	16.5	16.8, 5.70
W8	W-HPC-1:5, SDS	7.96 ± 0.1	25.0 ± 0.9	56.4 ± 0.5	16.5	16.8, 4.37
W9	W-VA64-1:5, SDS	3.16 ± 0.1	10.0 ± 0.5	25.5 ± 0.9	16.5	16.9, 3.82
S1	S-Sol-1:5	4.58 ± 0.5	15.4 ± 0.5	38.3 ± 0.4	16.7	17.1, 2.02
S2	S-HPC-1:5	5.31 ± 0.4	25.7 ± 0.6	66.8 ± 0.6	16.7	16.9, 5.23
S3	S-VA64-1:5	3.74 ± 0.7	18.7 ± 0.6	36.9 ± 1.0	16.7	17.0, 3.41

^a^ Ratios in the formulations refer to the drug:polymer mass ratio. ^b^ % *w*/*w* with respect to the total weight of the solid content.

**Table 5:** Temperatures–enthalpy values of various thermal events observed in the DSC traces and crystallinity estimated from the XRPD diffractograms.

ID	Formulation	*T*_g_ (°C)	*T*_m_ (°C)	Δ*H*_f_ (J/g)	% Crystallinity
AR	As-received ITZ	—	171	70.9	100
W1	W-AR-Sol-1:5	—	161	5.79	87.1
W2	W-Sol-5:1	—	162	34.4	83.6
W3	W-Sol-1:3	—	145	3.32	81.7
W4	W-Sol-1:5	—	136	1.64	79.8
W5	W-HPC-1:5	—	160	6.32	95.6
W6	W-VA64-1:5	—	141	7.46	92.7
W7	W-Sol-1:5, SDS	—	130	1.35	69.7
W8	W-HPC-1:5, SDS	—	159	4.91	84.9
W9	W-VA64-1:5, SDS	—	131	7.21	82.7
S1	S-Sol-1:5	72.0	—	—	—
S2	S-HPC-1:5	58.1	158	0.14	—
S3	S-VA64-1:5	100.9	—	—	—

**Table 6:** Properties of 0.1 N aq. solution and 0.1 N aq. HCl solutions of stabilizers and wetting effectiveness factor determined using the modified Washburn method.

Liquid	*η*, (cP)	*ρ*, (g/mL)	*γ*, (mN/m)	Slope, (g^2^/s) ^a^	*R* ^2^	cos *θ*_ss_/cos *θ*_w_	log(cos *θ*_ss_/cos *θ*_w_)
0.1 N HCl	0.89	1.00	72.0	3.5 × 10^−5^	0.988	1	0
Sol	8.85	1.01	41.1	2.3 × 10^−3^	0.998	1120	3.05
HPC	22.3	1.01	37.9	1.1 × 10^−3^	0.998	1470	3.17
VA64	5.16	1.01	39.9	4.9 × 10^−3^	0.998	1440	3.16
Sol–SDS	13.3	1.01	39.3	2.3 × 10^−3^	0.995	1760	3.25
HPC–SDS	27.5	1.01	35.5	1.5 × 10^−3^	0.999	2630	3.42
VA64–SDS	6.29	1.01	37.6	5.3 × 10^−3^	0.999	2010	3.30

^a^ See Section S1 of Supplementary Material for calculation of the slope.

## Data Availability

Data are contained within the article and its supplementary materials.

## Supplementary Materials

The following supporting information can be downloaded at: https://www.mdpi.com/article/10.3390/nano13172419/s1, Section S1: Details of the Methods Used for Drug Wettability Measurements; Section S2: XRPD of SDS and As-received Itraconazole (ITZ); Section S3: DSC Thermograms of the Physical Mixtures; Figure S1: Temporal evolution of the squared liquid mass penetrated into a packed bed of as-received ITZ particles for ITZ-saturated 0.1 N HCl solution and various ITZ-saturated 0.1 N HCl solutions of 12.5% HPC/Sol/VA64 with or without 0.125% SDS; Figure S2: X-ray diffractograms of as-received ITZ and SDS powders; Figure S3: DSC thermograms of the physical mixtures (PMs) of ITZ–HPC/Sol/VA64 with 1:5 drug:polymer mass ratio with and without SDS; Table S1: Thermal properties of the ITZ–HPC/Sol/VA64 physical mixtures (PMs) at 1:5 drug:polymer ratio with or w/o SDS. References [28,36,59,60,61] are cited in supplementary materials.
