# Supplementary material for: Preparation and Characterization of Spray-Dried Hybrid Nanocrystal–Amorphous Solid Dispersions (HyNASDs) for Supersaturation Enhancement of a Slowly Crystallizing Drug
Source: Nanomaterials (Basel). 2023 Aug 25;13(17):2419. doi: 10.3390/nano13172419 (PMC10490532; doi:10.3390/nano13172419)
Supplement: Supplementary file 1 [file nanomaterials-13-02419-s001.zip › nanomaterials-2554774-supplementary.pdf]

nanomaterials
MDPI

Supplementary Materials

Preparation and Characterization of Spray-Dried Hybrid Nanocrystal–Amorphous Solid Dispersions (HyNASDs) for Supersaturation Enhancement of a Slowly Crystallizing Drug

Mahbubur Rahman[1], Keanu Radgman[1], James Tarabokija[1], Stephanie Ahmad[1], Ecevit Bilgili[1*]

[1] Otto H. York Department of Chemical and Materials Engineering New Jersey Institute of Technology, Newark, NJ, United States
* Correspondence: bilgece@njit.edu

S1. Details of the Methods Used for Drug Wettability Measurements

Penetration of a liquid into a packed powder bed of a drug inside a cylindrical column allows for the measurement of the drug powder wettability, based on the Washburn method [1,2]. The method presented here was adapted from Bilgili et al. [3] and Li et al. [4]. In the current study, liquids and powder refer to ITZ (itraconazole)-saturated 0.1 N aq. HCl solution of 12.5% Sol/HPC/VA64 with or w/o 0.125% SDS and as-received ITZ powder, respectively. All percentages are (% w/w) with respect to the 0.1 N aq. HCl solution. This polymer concentration was selected following the polymer concentration in the suspension-based (W) and solution-based (S) formulations as stated in Table 1 of the main text. The stabilizer solutions and HCl solution were saturated with itraconazole (ITZ) and stirred overnight. After overnight stirring, the saturated solutions were used for further characterization.

S1.1. Apparent shear viscosity of the solutions

The apparent shear viscosity of the ITZ-saturated acid solutions (0.1 N HCl) of the stabilizers was measured using an R/S Plus Rheometer (Brookfield Engineering, Middleboro, MA, USA) with a water jacket assembly Lauda Eco (Lauda-Brinkmann LP, Delran, NJ, USA). A coaxial cylinder (CC40) was used to impart a controlled shear rate from 0 to 1000 1/s for 60 s. The temperature of the jacket was kept constant at 25 ± 0.5 °C. The raw data were analyzed using the Rheo 3000 software (Brookfield Engineering, Middleboro, MA, USA) of the equipment to obtain the apparent shear viscosity as a function of the shear rate. The apparent shear viscosity at ~100 1/s, a representative low shear rate value, was used in the calculations. The viscosity 0.1 N HCl solution was considered to be same as of water and the value was taken from Korson et al. [5].

S1.2. Surface tension of the solutions

The surface tension of the ITZ-saturated aq. HCl solution and the ITZ-saturated aq. HCl solutions of the stabilizers were measured using Attension Sigma 700 (Biolin Scientific, Linthicum, MD, USA). The Attention software calculates surface tension from force measurements of the interaction of a probe (Wilhelmy plate) at the boundary between air and the liquid.

S1.3. Drug wettability with the solutions

Attension Sigma 700 set-up (Biolin Scientific, Linthicum, MD, USA) was used to study the penetration of ITZ-saturated aq. HCl solution and ITZ-saturated aq. HCl solutions of the stabilizers into a packed powder bed of ITZ inside a cylindrical column and determine the ITZ wettability, based on the modified Washburn method. The assembly consists of a sample holder in the form of a cylindrical metallic tube with small holes at the bottom as well as a hook at the top of the cover equipped with screw threads. About 0.8 g of ITZ powder was packed uniformly into the tube before each measurement. A filter paper was placed at the perforated end of the sample holder to support the ITZ powder sample. A petri dish containing 0.1 N aq. HCl solution/0.1 N aq. HCl solution of the stabilizers (polymer/polymer–SDS) was placed below the perforated end of the holder on the mechanical platform.

Upon contact of the sample holder with the liquid, the liquid penetrated the ITZ powder bed, while Attension Sigma 700 recorded the mass M of the liquid penetrated as a function of time T. The cosine of the contact angle θ for the ITZ-saturated HCl solutions of the stabilizers and drug can be determined using the modified Washburn equation, which provides a relationship between the liquid penetration rate and contact angle via $M^2 = \left(\frac{C\rho^2\gamma\cos\theta}{\eta}\right)T$, where η, ρ,

and γ stand for the viscosity of the liquid, density of the liquid, and surface tension of the liquid, respectively. C is a characteristic parameter of the powder sample, which could have been determined independently using a complete wetting liquid such as hexane, heptane, etc. Since the same drug powder (ITZ) was used as the powder sample and C depends only on powder packing–size, C remained invariant for the different liquids studied here. This allows us to calculate the ratio of $\cos\theta_{ss}/\cos\theta_w$ as a wetting effectiveness factor from the slopes of M^2 vs. T for the aq. HCl solution and the aq. HCl solutions of the stabilizers. Here, θ_{ss} is the contact angle between ITZ and the aq. polymer–SDS stabilizer in HCl solution and θ_w is the contact angle between ITZ and the aq. HCl solution. The wettability enhancement upon the use of different stabilizers (polymer/polymer–SDS) on the wetting of ITZ particles can be assessed by using this ratio, taking the wettability by HCl solution as a basis for comparison.

Experimental liquid penetration data (M^2 vs. T) for various liquids are presented in Figure S1. The slope of the modified Washburn equation, i.e., $\frac{C\rho^2\gamma\cos\theta}{\eta}$, was obtained by fitting the linear region of the liquid penetration curve. The initial ~20 s was not considered due to transient behavior; data points that deviated from the linear region, which may correspond to structural changes in the bed, were excluded. The modified Washburn equation fitted the data well ($R^2 \geq 0.988$). Using the slope for the 0.1 N aq. HCl solution alone and the different stabilizers in the aq. HCl solution, $\cos\theta_{ss}/\cos\theta_w$ was calculated. The viscosity, surface tension, and calculated wetting effectiveness factor are reported in Table 5 of the main text.

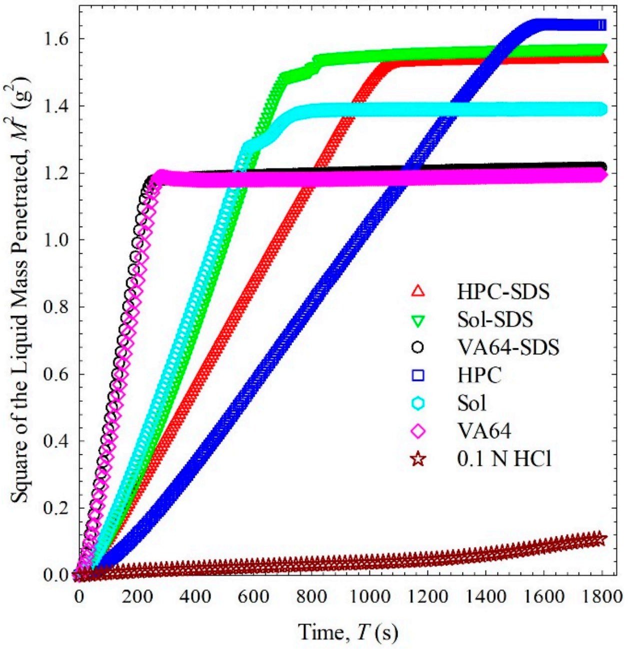

Figure S1. Temporal evolution of the squared liquid mass that penetrated ia packed bed of ITZ particles for ITZ-saturated 0.1 N aq. HCl solution and various ITZ-saturated 0.1 N aq. HCl solutions of 12.5% HPC/Sol/VA64 with or without 0.125% SDS.

S2. XRPD of SDS and As-received Itraconazole (ITZ)

Three intense non-overlapping peaks were detected at diffraction angles of 5.6°, 6.8°, and 8.3° in the SDS diffractogram (Figure S2, the peaks shown in the orange dashed rectangle). We used four intense non-overlapping peaks at diffraction angles of 17.5°, 17.9°, 20.3°, and 23.4° in the ITZ diffractogram (the peaks shown in the green dashed rectangle), which do not interfere with the characteristic peaks of SDS, in order to calculate ITZ crystallinity. It should be noted that as SDS is a minor component of the spray-dried powders (~0.8% w/w), its peaks were not as intense as the ITZ peaks in the diffractograms of the PMs (refer to Figure 1b of the main text). However, these characteristic peaks of SDS disappeared in W7–W9 samples (milled–spray-dried powders). During spray drying of the milled suspensions, which contained 0.125% (w/v) SDS dissolved in deionized water, a small amount of SDS was the most likely to be molecularly dispersed in the polymeric matrix owing to polymer–SDS interactions; thus, its crystallinity was not detected by XRPD.

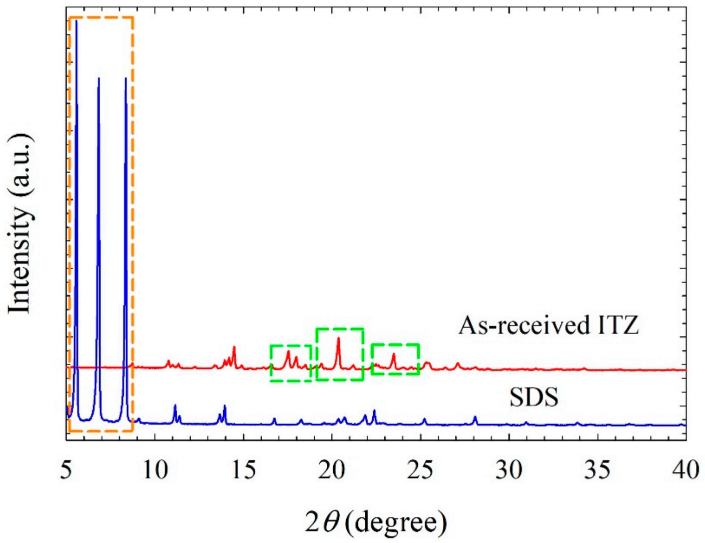

Figure S2. X-ray diffractograms of as-received ITZ and SDS powders.

S3. DSC Thermograms of the Physical Mixtures

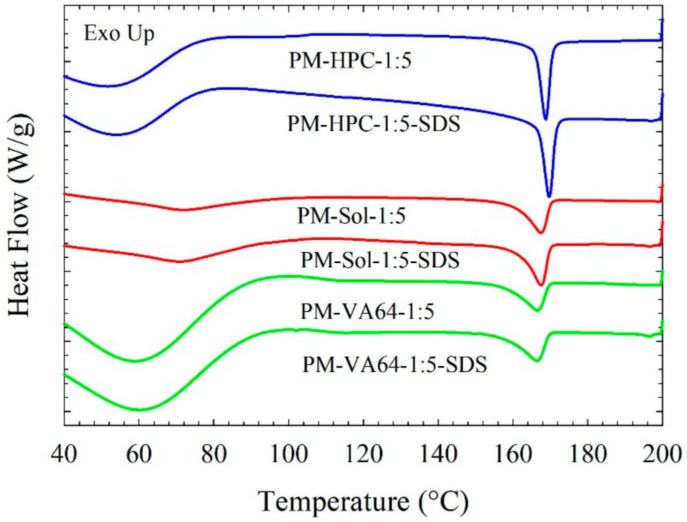

Figure S3. DSC thermograms of the physical mixtures (PMs) of ITZ–HPC/Sol/VA64 with 1:5 drug:polymer mass ratios with and without SDS.

Table S1. Thermal properties of the ITZ–HPC/Sol/VA64 physical mixtures (PMs) at 1:5 drug:polymer ratios with or w/o SDS.

Formulation[a]	T_g (°C)[b]	T_m (°C)[b]	ΔH_f (J/g)[b]	ΔT_m (°C)[b]
PM-HPC-1:5	57.5	169	10.7	2
PM-HPC-1:5, SDS	58.4	170	10.0	1
PM-Sol-1:5	71.6	167	7.14	4
PM-Sol-1:5, SDS	72.8	167	11.1	4
PM-VA64-1:5	102.6	166	8.25	5
PM-VA64-1:5, SDS	97.8	166	8.03	5

[a] Sol denotes Soluplus; the ratios refer to the drug:polymer mass ratios.

[b] T_m stands for melting point temperature, T_g stands for glass transition, ΔT_m stands for melting point depression, and ΔH_f stands for fusion enthalpy.

References

1. Washburn, E.W. The dynamics of capillary flow. *Phys. Rev.* **1921**, *17*, 273–283.
2. Hołownia, D.; Kwiatkowska, I.; Hupka, J. An investigation on wetting of porous materials. *Physicochem. Prob. Miner. Process.* **2008**, *42*, 251–262.
3. Bilgili, E.; Rahman, M.; Palacios, D.; Arevalo, F. Impact of polymers on the aggregation of wet-milled itraconazole particles and their dissolution from spray-dried nanocomposites. *Adv. Powder Technol.* **2018**, *9*, 2941–2956.
4. Li, M.; Ioannidis, N.; Gogos, C.; Bilgili, E. A comparative assessment of nanocomposites vs. amorphous solid dispersions prepared via nanoextrusion for drug dissolution enhancement. *Eur. J. Pharm. Biopharm.* **2017**, *119*, 68–80.
5. Korson, L.; Drost-Hansen, W.; Millero, F.J. Viscosity of water at various temperatures. *J. Phys. Chem.* **1969**, *73*, 34–39.
